# The application of subcapsular saline injection to preserve the parathyroid gland during thyroidectomy

**DOI:** 10.1016/j.heliyon.2023.e21543

**Published:** 2023-11-02

**Authors:** Li Zhou, Miao Zhang, Danqin Xu, Jingjing Shi, Gang Pan, Yu Zhang, You Peng

**Affiliations:** a Department of Surgical Oncology, Affiliated Hangzhou First People's Hospital, Zhejiang University School of Medicine, Hangzhou, China; b The Fourth Clinical Medical College, Zhejiang Chinese Medical University, Hangzhou, China

**Keywords:** Thyroidectomy, Hypoparathyroidism, Hypocalcemia, Subcapsular saline injection

## Abstract

Objective: To evaluate the clinical effect of subcapsular saline injection (SCASI) after total thyroidectomy. Methods: A total of 77 patients who underwent total thyroidectomy in our hospital from January 2020 to December 2021 were selected and divided into the SCASI group (n = 43) and the non-SCASI group (n = 34). The general clinical data of the patients were collected, and serum parathyroid hormone (PTH) and serum calcium levels were determined preoperatively, on the 1st postoperative day, and at 1 and 6 months after the operation. These data were compared between groups. Results: There was no significant difference in postoperative complications between the two groups. The PTH and serum calcium levels in the SCASI group were significantly higher than those in the non-SCASI group on the 1st postoperative day (t = 2.340, 5.208, both P < 0.05), and the PTH levels in the SCASI group at 1 month after the operation were higher than those in the non-SCASI group (t = 2.141, P < 0.05). In addition, the proportion of transient and permanent hypoparathyroidism in the SCASI group was significantly decreased (χ^2^ = 3.920, 3.948, P < 0.05). Conclusion: Total thyroidectomy requires high surgical precision, and SCASI can reduce the incidence of temporary and permanent hypoparathyroidism.

## 1 Introduction

Hypoparathyroidism is a common complication of total thyroidectomy. Temporary hypoparathyroidism occurs in 5.8 %–60 % of patients undergoing total thyroidectomy, and permanent hypoparathyroidism occurs in 0.9 %–6.3 % of patients undergoing total thyroidectomy [1]. Symptoms of hypoparathyroidism include perioral numbness as well as tingling or numbness in the extremities; furthermore, tetany may occur in severe cases, requiring large doses of calcium and vitamin D supplements to maintain serum calcium levels, thereby significantly reducing patients’ quality of life [2]. Multiple previous studies have aimed to examine the preservation of parathyroid function in thyroid surgery by preserving branches of the inferior thyroid artery; performing autotransplantation of the parathyroid; and identifying parathyroid glands using indocyanine green, carbon nanoparticles, or gamma probes [3,4]. Due to the lack of improvements in surgical strategies, preserving parathyroid function is technically challenging and largely depends on the experience of the surgeon. Compared with other techniques, subcapsular saline injection (SCASI) has been shown to enable a better parathyroid blood supply and enhanced in situ preservation of the parathyroid gland. Here, we report the protective effects of SCASI on A1-or A2-type parathyroid during total thyroidectomy based on the 2015 Chinese Expert Consensus on Parathyroid Protection in Thyroidectomy.

## 2 Methods

### 2.1 Patients

Patients who underwent total thyroidectomy for papillary thyroid carcinoma (PTC) from January 2020 to December 2021 were retrospectively evaluated. All patients were operated on by the same surgeon and classified into two groups based on whether SCASI was used during the procedure: patients with or without SCASI. The following demographic and clinical information of all patients was collected: age, sex, body mass index (BMI), comorbidities, operative time, tumor size, pathology, central neck dissection, Hashimoto thyroiditis (HT), postoperative complications and duration of follow-up (months). Serum parathyroid hormone (PTH) and calcium (Ca) were measured preoperatively, on postoperative day (POD) 1, and at 1 and 6 months after surgery. Transient and permanent hypoparathyroidism were defined as PTH levels lower than 10 ng/mL on POD 1 and lower than 15 ng/mL at 6 months after surgery, respectively [5].

### 2.2 Anatomic classification of parathyroid glands

According to the 2015 Chinese Expert Consensus on Parathyroid Protection in Thyroidectomy [6], the parathyroid glands are divided into type A and type B based on the positional relationship between the thyroid and parathyroid as well as the difficulty of retaining the gland in situ (Table 1).

**Table 1:** Anatomical classification of parathyroid glands.

Type A		The compact type, the thyroid and parathyroid glands are located close together, difficult to retain them in situ.
	Type A1	The parathyroid glands are attached to the surface of the thyroid.
	Type A2	The parathyroid glands are partially or fully embedded in the thyroid, but outside the natural capsule.
	Type A3	The parathyroid glands are completely within the thyroid and inside the natural capsule, unlike type A2.
Type B		The non-compact type, there is a natural gap between the thyroid and the parathyroid, easier to retain in situ.
	Type B1	Peripheral thyroid type, including all type B parathyroid glands except B2 and B3.
	Type B2	Intra-thymus type, in which the parathyroid glands are located in the thymus.
	Type B3	blood supply from vessels of the thymus or mediastinum.

### 2.3 Subcapsular saline injection method

**Figure 1:**
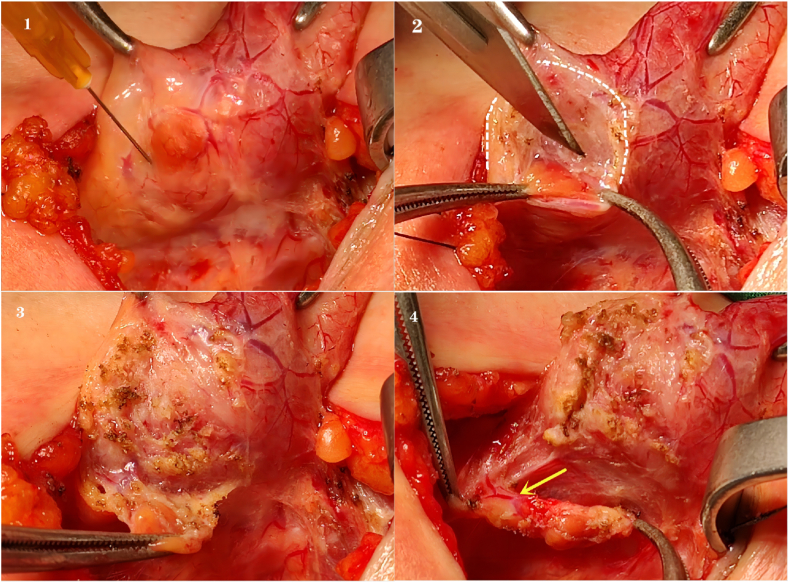
A. Surgical image of the subcapsular saline injection method.

**Figure 2:**
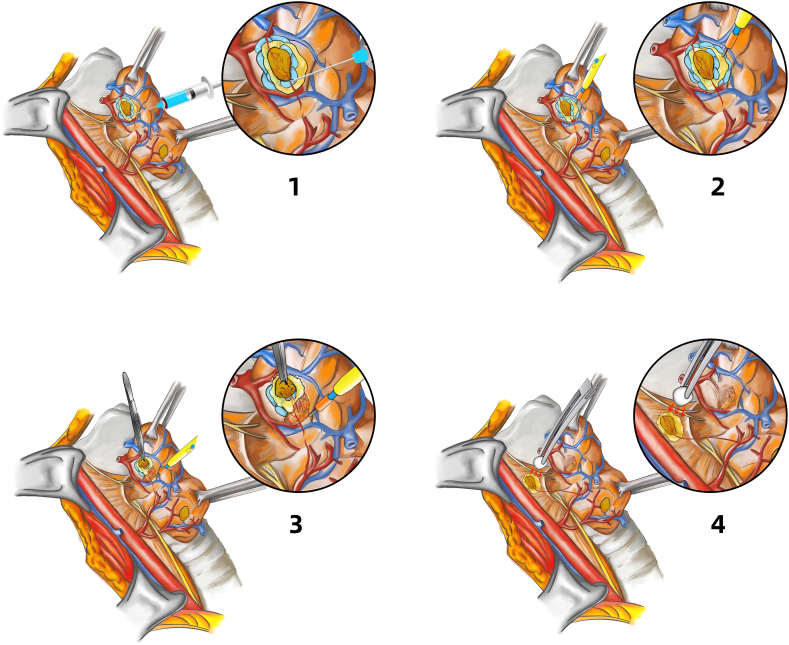
B. Schematic diagram of the subcapsular saline injection method. A1, B1. Normal saline (2–3 mL) was injected into the subcapsular layer of the thyroid with a 26-gauge needle. A2, B2. Anatomical plane of the inflated subcapsular layer along the thyroid margin. A3, B3. Parathyroid glands after dissection along the indicated anatomical plane. A4, B4. Location of the parathyroid gland and its feeding vessels.

After general anesthesia, the patient was placed in a supine position with slight neck extension. A horizontal skin incision was made at the level of the suprasternal notch, and conventional flap dissection was performed. Then, isthmectomy was performed with the FOUCUS ultrasonic scalpel after the midline division of the strap muscles. Following lateral dissection, the thyroid and the parathyroids were exposed by medial traction of the thyroid gland. A 26-gauge needle attached to a 5-mL syringe was used to inject the subcapsular layer of the thyroid gland around the parathyroids with 2–3 mL of 0.9 % normal saline (Figure 1, Figure 2). Then, the inflated subcapsular layer along the thyroid margin was electrocuted (Figure 1, Figure 2). Once the parathyroid gland became visible, mini-incision meticulous dissection techniques were carefully performed (Figure 1, Figure 2), to preserve the parathyroid gland and its blood supply (Figure 1, Figure 2).

### 2.4 Statistical analysis

All statistical analyses were performed using SPSS 25.0 software (SPSS Institute, Chicago, IL). Continuous variables were expressed as the means and standard deviations, and categorical data were expressed as counts and percentages. For continuous variables, differences between groups were compared with independent samples t tests. For categorical data, differences between groups were compared using chi-square tests. Results were considered statistically significant at two-sided p values < 0.05.

## 3 Results

A total of 77 patients were divided into two groups: the SCASI group consisted of 43 patients (8 men, 35 women) with a mean age of 51.62 ± 5.44 years, while the non-SCASI group consisted of 34 patients (5 men, 29 women) with a mean age of 52.92 ± 4.74 years. There was no significant difference in age, sex, BMI, comorbidities, operative time, tumor size, pathology, central neck dissection, HT or postoperative complications (Table 2).

**Table 2:** Characteristics of the patients with or without subcapsular saline injection.

	Non-SCASI（n = 34）	SCASI（n = 43）	χ^2^/t value	P value
Age, years	52.92 ± 4.74	51.62 ± 5.44	1.101	0.274
Gender ratio, male:female	5/29	8/35	0.206	0.650
BMI(kg/m^2^)	22.97 ± 2.55	22.45 ± 2.49	0.90	0.371
Comorbidities, n (%)				
Hypertension	3（8.822）	2（4.65）	0.544	0.461
Diabetes	2（5.88）	1（2.32）	0.642	0.423
Tumor size, cm	1.53 ± 0.88	1.56 ± 0.79	0.157	0.876
Operative time, hour	1.18 ± 0.31	1.28 ± 0.49	1.036	0.303
Central neck dissection, n (%)			0.030	0.862
Bilateral CND	22（64.71）	27（62.8）		
Ipsilateral CND	12（35.29）	16（37.20）		
Hashimoto thyroiditis, n (%)	3（8.82）	4（9.30）	0.005	0.942
Postoperative complications, n (%)				
Dysphagia	2（5.88）	2（4.7）	0.058	0.809
Hoarseness	1（2.94）	2（4.7）	0.148	0.700

The serum PTH and calcium levels of the patients in the SCASI group were significantly higher on postoperative day 1 than those of the patients in the non-SCASI group (t = 2.340, 5.208, both P < 0.05). Furthermore, the serum PTH levels of the SCASI group at 1 month after the operation were significantly higher than those of the non-SCASI group. In addition, the patients in the SCASI group were significantly less likely to develop transient or permanent hypoparathyroidism than those in the non-SCASI group (χ2 = 3.920, 3.948, both P＜0.05) (Table 3).

**Table 3:** The serum parathyroid hormone (PTH) and calcium (Ca) of the two groups.

	Non-SCASI（n = 34）	SCASI（n = 43）	χ^2^/t value	P value
PTH, pg/mL				
Preoperative	48.22 ± 7.25	47.30 ± 8.03	0.521	0.604
Postoperative day 1	17.73 ± 5.66	21.72 ± 8.57	2.340	0.022
1month after surgery	21.23 ± 4.32	24.30 ± 7.42	2.141	0.035
6 months after surgery	32.11 ± 5.11	33.51 ± 4.82	1.232	0.222
Transient hypoparathyroidism, n (%)	14（41.18）	6（13.95）	3.920	0.048
Permanent hypoparathyroidism, n (%)	3（8.82）	0（0.0）	3.948	0.047
Ca, mmol/mL				
Preoperative	2.39 ± 0.16	2.45 ± 0.25	1.216	0.228
Postoperative day 1	1.22 ± 0.11	1.38 ± 0.15	5.208	＜0.001
1month after surgery	2.14 ± 0.16	2.09 ± 0.19	1.228	0.223
6 months after surgery	2.50 ± 0.14	2.51 ± 0.11	0.351	0.727

## 4 Discussion

Although the vascularity of the parathyroid gland and thyroid gland has been determined, it remains difficult for surgeons to preserve the blood supply of the parathyroid gland during surgery. Even with visualization techniques in endoscopic surgery, it is difficult to protect the blood supply of the parathyroid in order to decrease the incidence of hypoparathyroidism [3,4]; furthermore, this observation needs to be confirmed in larger prospective studies. SCASI was recently reported to be an outstanding method for preserving the parathyroid glands in situ [5]. The aim of this study was to determine the clinical effect of SCASI on parathyroid glands in patients undergoing total thyroidectomy.

No significant differences in other postoperative complications were observed between the two groups; however, a significantly lower rate of transient hypoparathyroidism was observed in the SCASI group. More importantly, no patient in the SCASI group developed permanent hypoparathyroidism, while 3 patients in the non-SCASI group did develop permanent hypoparathyroidism and required calcium supplementation for long periods. Therefore, the results suggest that SCASI could significantly decrease the incidence of hypoparathyroidism after total thyroidectomy. The percentages of transient and permanent hypoparathyroidism reported herein were significantly lower than the rates in previous studies (5.8 %–60 % and 0.9 %–6.3 %); the decrease was a result of the application of meticulous capsular dissection techniques in the two groups, which can help preserve the parathyroid in situ and maintain its blood supply. Additionally, while previous studies have reported an association between central neck dissection (CND) and a higher rate of hypoparathyroidism, there was no difference between the status of CND in the two groups herein. In accordance with the 2015 Chinese Expert Consensus on Parathyroid Protection in Thyroidectomy, the parathyroid glands were classified as type A and type B based on the positional relationship between the thyroid and the parathyroid and based on the difficulty of preserving parathyroid glands in situ. Theoretically, the type B parathyroid is easier to preserve than the type A parathyroid, and we only performed the mini-incision meticulous dissection techniques to leave the parathyroid glands in situ. Type A parathyroid glands are situated close to the thyroid; therefore, it is difficult to preserve type A1 and A2 glands in situ, and it is not possible to preserve type A3 glands in situ. In this study, the proportion of type A glands in the SCASI group was higher than that in the non-SCASI group, but the rate of hypoparathyroidism was lower in the SCASI group. Therefore, we believe that SCASI contributes to the in situ preservation of types A1 and A2 parathyroid glands.

Due to the small size of parathyroid glands, they could easily be damaged by uncontrolled force or inappropriate movement. Then, the reduction in PTH may lead to permanent hypocalcemia that is difficult to treat and severely compromises a patient's quality of life. Intraoperative identification of parathyroid glands is always challenging for surgeons performing thyroid surgery. Fluorescence imaging techniques and angiography with indocyanine green and carbon nanoparticles could serve as alternative approaches, but they still cannot provide a clear visualization of the blood supply and are even associated with some adverse reactions to the stain [[7], [8], [9], [10]]. Endoscopic visualization thyroidectomy can accurately identify the position of parathyroid glands during surgery to avoid unintentional resection. Due to the anatomic features of parathyroid glands, visualization could be hampered easily. First, the blood supply of the parathyroid is very small (<1 mm in diameter), making it difficult to observe them in connective tissues [11]. Second, numerous anatomic variations may occur, although parathyroid glands receive their blood supply from tertiary branches from the inferior and superior thyroid arteries [12]. Finally, the lower parathyroid glands could be found anywhere from the inferior thyroid to the superior mediastinum because the precursor cells settle randomly during embryonic development, making the location inconsistent [13].

In contrast, SCASI can achieve fine stripping of the thyroid capsule, avoid ligation of the inferior thyroid artery if possible, and preserve the terminal branch feeding the parathyroid glands. This method has 3 advantages. First, saline injection can expand the space around the parathyroid and thus can widen the distance between the blood supply of the parathyroid and the surgical instruments, in turn minimizing surgical injury to the blood supply. Moreover, a saline pocket around the parathyroid gland may be generated after the injection to decrease the thermal or mechanical injury caused by energy devices such as electrotome and ultherapy [14]. Second, saline injection reduces the yellow color, thus enabling observers to distinguish parathyroid tissue from surrounding lymph nodes and adipose tissue. Third, SCASI is easy to perform and does not incur additional medical costs. The deficiency is that although SCASI decreases the rate of hypoparathyroidism, the surgical procedure is complex and technically demanding [15] due to the anatomy and distribution heterogeneity of the blood supply of parathyroid glands. For the parathyroid glands that are considered to be devascularized, we conducted parathyroid autotransplantation.

The main limitation of this study lies in the fact that it is a single-center, retrospective cohort study with a rather small sample size; therefore, the conclusions still need to be confirmed. The non-SCASI group underwent surgery earlier than the SCASI group, which led to a lack of temporal concurrency. Finally, SCASI is a technically demanding procedure, and more in-depth studies are needed before this method is widely applied. In conclusion, the exact location and vascular structure of the parathyroid glands are difficult to determine by the naked eye, but the rate of transient and permanent hypoparathyroidism could be decreased by performing SCASI.

## Ethical approval

All procedures performed in studies involving human participants were in accordance with the ethical standards of the institutional and/or national research committee and with the 1964 Helsinki Declaration and its later amendments or comparable ethical standards. The study was approved by the Ethics Committee of Affiliated Hangzhou First People's Hospital, Zhejiang University School of Medicine. Informed consent was obtained from all participants in the study.

## Data availability statement

The date that support the findings of this study are available from the corresponding author upon reasonable requst.

## Funding

This work was supported by the basic public welfare research project of Zhejiang province (LGF22H070008); Zhejiang medical and health science and technology Project, No. 2021KY850; Hangzhou medical science and technology project (OO20191087).

## CRediT authorship contribution statement

**Li Zhou:** Writing – review & editing, Writing – original draft, Formal analysis, Data curation, Conceptualization. **Miao Zhang:** Writing – original draft, Formal analysis, Data curation, Conceptualization. **Danqin Xu:** Formal analysis, Data curation. **Jingjing Shi:** Formal analysis, Data curation. **Gang Pan:** Software. **Yu Zhang:** Software. **You Peng:** Writing – review & editing, Visualization, Conceptualization.

## Declaration of competing interest

The authors declare that they have no known competing financial interests or personal relationships that could have appeared to influence the work reported in this paper.
